# Diagnostic utility of brain MRI volumetry in comparing traumatic brain injury, Alzheimer disease and behavioral variant frontotemporal dementia

**DOI:** 10.1186/s12883-024-03844-4

**Published:** 2024-09-11

**Authors:** Cyrus A. Raji, Somayeh Meysami, Verna R. Porter, David A. Merrill, Mario F. Mendez

**Affiliations:** 1 Mallinckrodt Institute of Radiology, Washington University, St. Louis, MO USA; 2 Department of Neurology, Washington University, St. Louis, MO USA; 3 Pacific Brain Health Center, Pacific Neuroscience Institute and Foundation, Santa Monica, CA USA; 4 Saint John’s Cancer Institute at Providence Saint John’s Health Center, Santa Monica, CA USA; 5 Providence Saint John’s Health Center, Santa Monica, CA USA; 6 Department of Psychiatry and Biobehavioral Sciences, David Geffen School of Medicine, University of California Los Angeles, Los Angeles, CA USA; 7 Department of Neurology, David Geffen School of Medicine, University of California Los Angeles, Los Angeles, CA USA; 8 Greater Los Angeles VA Healthcare System, Los Angeles, CA USA

**Keywords:** MRI volumetry, Traumatic brain injury, Alzheimer disease, Dementia

## Abstract

**Background:**

Brain MRI with volumetric quantification, MRI volumetry, can improve diagnostic delineation of patients with neurocognitive disorders by identifying brain atrophy that may not be evident on visual assessments.

**Objective:**

To investigate diagnostic utility of MRI volumetry in traumatic brain injury (TBI), early-onset Alzheimer disease (EOAD), late-onset Alzheimer disease, and behavioral variant frontotemporal dementia (bvFTD).

**Method:**

We utilized 137 participants of TBI (*n* = 40), EOAD (*n* = 45), LOAD (*n* = 32), and bvFTD (*n* = 20). Participants had 3D T1 brain MRI imaging amendable to MRI volumetry. Scan volumes were analyzed with Neuroreader. One-way ANOVA compared brain volumes across diagnostic groups. Discriminant analysis was done with leave-one-out cross validation on Neuroreader metrics to determine diagnostic delineation across groups.

**Result:**

LOAD was the oldest compared to other groups (F = 27.5, *p* < .001). There were no statistically significant differences in sex (*p* = .58) with women comprising 54.7% of the entire cohort. EOAD and LOAD had the lowest Mini-Mental State Exam (MMSE) scores compared to TBI (*p* = .04 for EOAD and *p* = .01 for LOAD). LOAD had lowest hippocampal volumes (Left Hippocampus F = 13.1, Right Hippocampus F = 7.3, *p* < .001), low white matter volume in TBI (F = 5.9, *p* < .001), lower left parietal lobe volume in EOAD (F = 9.4, *p* < .001), and lower total gray matter volume in bvFTD (F = 32.8, *p* < .001) and caudate atrophy (F = 1737.5, *p* < .001). Areas under the curve ranged from 92.3 to 100%, sensitivity between 82.2 and 100%, specificity of 78.1-100%. TBI was the most accurately delineated diagnosis. Predictive features included caudate, frontal, parietal, temporal lobar and total white matter volumes.

**Conclusion:**

We identified the diagnostic utility of regional volumetric differences across multiple neurocognitive disorders. Brain MRI volumetry is widely available and can be applied in distinguishing these disorders.

**Supplementary Information:**

The online version contains supplementary material available at 10.1186/s12883-024-03844-4.

## Introduction

The differentiation of chronic neurocognitive disorders, particularly in the absence of accessible biomarkers, can be a major diagnostic challenge. Neurodegenerative diseases such as Alzheimer disease (AD) and behavioral variant frontotemporal dementia (bvFTD) affect millions of people worldwide [1], and traumatic brain injury (TBI) accounts for millions of emergency department visits and hospitalizations each year [2]. These conditions share common symptoms such as memory loss, language problems, and cognitive impairment and may be difficult to accurately distinguish [3]. Yet, the accurate diagnosis of these disorders is critical for proper treatment and management. Currently, clinical evaluation and neuropsychological testing are the standard diagnostic methods. The development of fluid biomarkers for AD, such as plasma amyloid [4] and tau [5], and blood and CSF biomarkers for TBI and bvFTD, hold great promise for the future, but clinicians need a currently accessible method for helping diagnosis these disorders.

A more quantitative analysis of magnetic resonance imaging (MRI) of the brain may contribute greatly to the diagnosis of these disorders. Brain MRI is widely available, with close to 12,000 scanners in the United States [6]. Magnetic Resonance imaging (MRI) volumetry is a computer based technique used to measure the volume of brain structure and to determine abnormality by comparison to a normal database or control group and has the most systematic reviews compared to other radiology techniques [7]. Manual volumetry, while considered the gold standard, is time-consuming and subject to user variability [8]. MRI volumetry builds upon other brain volume estimation methods from boundary shift integral [9] to voxel based morphometry [10] and has resulted in multiple fully automated FDA cleared algorithms used in clinical practice [11, 12]. However, very few of these clinical programs have data on their comparative performance across multiple diagnoses. One study did show that use of MRI volumetry with a software called QReport improved diagnostic interpretation of brain MRI scans from normal cognition, AD and FTD. Sensitivity with MRI volumetry improved diagnostic visual interpretations alone from 71.5 to 82.2% (*p* = .01, Cohen’s D = 1.03); specificity went from 78.5 to 72.3% but this was not statistically significant. Finally, accuracy improved from 71.1 to 80% (*p* = .02, Cohen’s D = 4) [13].

Additional work is required to optimally refine the use of clinical MRI volumetry software programs towards delineating common causes of cognitive decline from one another by identifying atrophic structures that differ across such disorders. This work therefore aims to evaluate the diagnostic utility of an FDA cleared MRI volumetry program in delineating the common chronic neurocognitive disorders of Alzheimer disease (both late-onset [LOAD] and early-onset [EOAD], traumatic brain injury (TBI), and behavioral variant frontotemporal dementia (bvFTD). Our previous work has suggested specific atrophy patterns using such software in each of these conditions [14–16]. We hypothesize this approach would provide high performance diagnostic utility based on specific regions for a given diagnosis, for example the hippocampus in LOAD [17].

## Methods

### Research participants

This was a retrospective study conducted in adherence to the STARD guidelines for diagnostic accuracy studies [18]. Eligible research participants presented to specialty dementia clinics and underwent extensive evaluations under UCLA institutional review board (IRB) approved studies (IRB#16–000496, IRB#16-001491, and IRB#10-001097) with related informed consent obtained from both participants and their caregivers. Each of the 137 participants included in this study had one of four diagnoses related to cognitive decline: TBI, LOAD, EOAD, or bvFTD. Participants in the entire study were 65.8 ± 12.6 years with an age range of 25–95 years of age. Mean MMSE for the entire cohort was 22.2 ± 5.9 and little over half of the total cohort were women (75/137, 54.7%). These patients presented to memory or behavioral neurology clinics and were diagnosed using established clinical criteria for these disorders by clinical specialists in behavioral neurology (Mario F. Mendez, Verna R. Porter) and geriatric psychiatry (David A. Merrill). All of these cohorts and the related diagnostic approaches have been fully described in previous published literature [14–16].

### Structural MR neuroimaging

The quantified MRI brain volumes were done for this study and not as part of the initial diagnostic evaluation. Each individual underwent brain MRI including a 3D volumetric T1 sequence on either a 1.5 or 3.0 Tesla Siemens Scanner. The 3.0T protocol was on a Siemens MAGNETOM Trio MRI scanner with acquisition of high-resolution T1-weighted 3D MPRAGE sequences with the following parameters: 192 × 256 matrix and TR = 1,900 ms, TE = 4.38 ms, TI = 1,100 ms, flip angle 15°, voxel size of 1 × 1 × 1 mm3. The 1.5T protocol was done on a Siemens Avanto scanner on which an MPRAGE was also obtained with the following parameters: T1-weighted sequences (256 × 256 matrix; TR = 2000 ms; TE = 2.89 ms; TI = 900 ms; flip angle = 40°; voxel size = 1 × 1 × 1 mm3). Scans were then analyzed with Neuroreader, an FDA cleared volumetric program described in previous work [14, 19]. Using Neuroreader, a total of 45 brain structures were quantified including the hippocampus, lobar structures, subcortical regions (thalamus, caudate, etc.), ventral diencephalon, midbrain, ventricular, and white matter volumes with an atlas-based segmentation, also detailed previously [19]. Segmentation of structures with Neuroreader is done using a multi-atlas based approach [20] in which, for example with the hippocampus, the 10 atlased images with the highest normalized correlation coefficient to the input image are non-linearly registered to the input image using an inverse-consistent symmetric free form deformation method [21]. From this segmentation, Neuroreader analysis generates the following results: (1) regional brain volume in milliliters; (2) region of interest volume as a fraction of measured total intracranial volume (Volume/mTIV), with mTIV being the sum of gray matter (GM), white matter (WM), and cerebrospinal fluid (CSF); (3) a proprietary estimation on the number of standard deviations from the normative database (NR index); (4) the number of standard deviations from the mean scaled between − 2 and + 2 (Z-score); and (5) percentile of comparison to the normative database (NR Percentile), which was drawn from cognitively normal controls from the Alzheimer Disease Neuroimaging Initiative (ADNI) [19]. No adverse events were reported in the clinical evaluations, or the brain MRI scans.

### Statistical analysis

All analyses were done in IBM SPSS (Version 27, IBM, Armonk, NY). A one-way ANOVA model was used to compare brain volumes across the diagnostic groups (TBI, bvFTD, EOAD, LOAD) to understand the range of lowest structural volumes or highest CSF containing areas per diagnostic group and post-hoc correction for multiple comparisons was done using Hocherg’s GT2 [22]. To determine if MRI structural metrics derived from the Neuroreader software program could delineate persons with TBI, bvFTD, EOAD, and LOAD from one another, we utilized discriminant analysis models with leave one out cross validation with separate models for (i) Model 1: Volume/mTIV (ii) Model 2: Volume (ml) (iii) Model 3: NR index (iv) Model 4: NR Percentile and (v) Model 5: Z-score. From each of these models, predicted probabilities of group membership derived from discriminant scores were inputted into ROC curve analysis to determine sensitivity, specificity, and accuracy. The model 3 ROC was redone with predicted probabilities in which for each region a NR index ≤ -2 cutoff was used. The model 4 ROC was also redone with the Neuroreader percentile ≤ 25th percentile for brain regions and Neuroreader percentile ≥ 75th percentile for CSF containing structures – the lateral ventricles. This approach was to determine if the results varied based upon the application of standard cutoffs that could simplify application of MRI volumetry to clinical practice. Automatic linear modeling was then used to select important predictors for each model. Separate one-way ANOVA compared age and MMSE across diagnostic groups and Chi-square evaluated group wise proportions of men and women.

## Results

Participant demographics and MMSE scores are shown in Table 1. In terms of age, the LOAD group was the oldest compared to other groups, with a statistically significant difference (F = 27.5, *p* < .001). No statistically significant differences were found regarding sex (*p* = .58), with women making up 54.7% of the entire cohort. Early-Onset Alzheimer’s Disease (EOAD) and LOAD groups had lower MMSE scores in comparison to the TBI group (*p* = .04 for EOAD and *p* = .01 for LOAD). Direct comparison of disease duration is difficult as the duration of time from TBI is not precisely known for all participants. However, the average disease duration across the neurodegenerative diseases, bvFTD, EOAD, and LOAD for this sample was estimated at 3.73 ± 2.1 and this was not statistically significant between these groups based upon prior work [15, 16]. While TBI severity was not available for this cohort, the time elapsed since trauma is as follows: Less than 6 months (10%), 6 months to less than 1 year (17.5%), 1 year or longer but less than 5 years (35%), 5 or more years (37.5%); there was no correlation with between time from injury and brain volumes [14].

The majority of participants (95/137, 69.3%) had 3.0T MRI. Supplemental Table 1 presents the ANOVA results for the lowest brain volume or largest CSF containing structure across various diagnoses, including TBI, bvFTD, EOAD, and LOAD. The results indicate that significant differences were observed across multiple brain regions for each diagnosis. For TBI, significant differences were found in white matter, brainstem, cerebellum, and frontal and parietal lobes. In the bvFTD group, gray matter, CSF, and several subcortical structures, such as the caudate, putamen, thalamus, ventral diencephalon, and pallidum, showed significant differences. The EOAD group exhibited significant differences in the left parietal lobe, while the LOAD group had significant differences in whole brain matter, hippocampus, temporal lobes, amygdala, lateral ventricles, occipital lobes, and pallidum. These findings indicate distinct patterns of brain volume alterations across different diagnoses, highlighting the importance of understanding the unique neuropathological characteristics of each condition.

**Table 1:** Participant demographics

	TBI (*n* = 40)	bvFTD (*n* = 20)	EOAD (*n* = 45)	LOAD (*n* = 32)	(t, F-value, Chi-square, *p*-value)
Age (mean, standard deviation)	67.7 ± 14.5	61.2 ± 10.9	59 ± 4.5	79.5 ± 7.8	F = 27.5, *p* < .001
Sex (M/F)	17/23	10/10	18/27	17/15	Chi-square = 1.94, *p* = .58
MMSE	24.1 ± 5.9	24.1 ± 4.9	19.9 ± 6.5	20.2 ± 6.4	F = 5.1, *p* = .002

*Abbreviations* TBI = Traumatic Brain Injury, bvFTD = behavioral variant frontotemporal dementia, EOAD = early onset Alzheimer disease, LOAD = late onset Alzheimer disease. MMSE = mini-mental state examination

### Model 1 Volume/measured total intracranial volume (mTIV) results

Discriminant Analysis for Model 1 (Volume/mTIV) showed an accuracy of 89.1% for the original grouped cases and 80.3% for cross-validated cases, in Table 2. The Automatic Linear Modeling method identified several predictor regions, with the highest predictor importance attributed to the right and left caudate, left ventral diencephalon, and brainstem, among others. The performance metrics for each diagnostic group showed high sensitivity and specificity values. The TBI group achieved 100% sensitivity, specificity, and area under the curve. The bvFTD group exhibited 95% sensitivity, 92.3% specificity, and a 98.3% area under the curve. The EOAD group showed 95.6% sensitivity, 91.3% specificity, and a 97.9% area under the curve. Lastly, the LOAD group had 90.6% sensitivity, 86.7% specificity, and a 95.4% area under the curve.

**Table 2:** Model 1 Volume/mTIV results

Classification method	% of Original grouped cases correctly classified		% of cross-validated cases correctly classified
Discriminant analysis	89.1%		80.3%
**Automatic linear modeling predictor region**			**Predictor importance**
Right caudate			0.14
Left caudate			0.13
Left ventral diencephalon			0.11
Brainstem			0.10
Right occipital lobe			0.10
CSF			0.10
Right parietal lobe			0.10
Left frontal lobe			0.09
Right frontal lobe			0.09
Gray matter			0.02
**Diagnostic group**	**Sensitivity**	**Specificity**	**Area under the curve**
TBI	100%	100%	100%
bvFTD	95%	92.3%	98.3%
EOAD	95.6%	91.3%	97.9%
LOAD	90.6%	86.7%	95.4%

Table 3 shows the Model 2 results for Discriminant Analysis and Automatic Linear Modeling. Discriminant Analysis achieved 89.8% accuracy for original grouped cases and 79.6% for cross-validated cases. Automatic Linear Modeling identified key predictor regions, such as right and left caudate and left cerebellum. High sensitivity and specificity were observed across diagnostic groups.

**Table 3:** Model 2 Volume (ml) results

Classification method	% of original grouped cases correctly classified		% of cross-validated cases correctly classified
Discriminant analysis	89.8%		79.6%
**Automatic linear modeling predictor region**			**Predictor importance**
Right caudate			0.12
Left caudate			0.11
Left cerebellum			0.10
Right thalamus			0.10
Left ventral diencephalon			0.10
Brainstem			0.10
CSF			0.10
Right parietal lobe			0.07
Left frontal lobe			0.06
Right frontal lobe			0.05
**Diagnostic group**	**Sensitivity**	**Specificity**	**Area under the curve**
TBI	100%	100%	100%
bvFTD	95%	91.3%	98.7%
EOAD	95.6%	90.2%	98.4%
LOAD	90.6%	90.5%	95.9%

For the NR index (Model 3) discriminant Analysis achieved 91.2% accuracy for original grouped cases and 76.6% for cross-validated cases. Automatic Linear Modeling identified key predictor regions, such as gray matter and frontal lobes. With the NR index cutoff results, TBI showed 97.5% sensitivity, 95.6% specificity, and a 99.7% area under the curve. The bvFTD group exhibited 95% sensitivity, 88.9% specificity, and a 98.2% area under the curve. EOAD had 91.1% sensitivity, 87% specificity, and a 92.3% area under the curve. LOAD displayed 84.4% sensitivity, 78.1% specificity, and a 92.3% area under the curve (see Tables 4).

**Table 4:** Model 3 NR index results

Classification method	% of original grouped cases correctly classified		% of cross-validated cases correctly classified
Discriminant analysis	91.2%		76.6%%
**Automatic linear modeling predictor region**			**Predictor importance**
Gray matter			0.15
Left frontal lobe			0.07
Right frontal lobe			0.07
White matter			0.06
Whole brain matter (gray matter + white matter)			0.06
Right ventral diencephalon			0.05
Left thalamus			0.05
Right thalamus			0.04
Right lateral ventricle			0.04
Right pallidum			0.04
**Diagnostic group**	**Sensitivity**	**Specificity**	**Area under the curve**
TBI	100%	100%	100%
bvFTD	100%	97.4%	99.7%
EOAD	93.3%	92.4%	97.2%
LOAD	93.8%	91.4%	96.8%
	**Sensitivity NR index cutoff ≤ -2**	**Specificity NR index cutoff ≤ -2**	**Area under the curve NR index cutoff ≤ -2**
TBI	97.5%	95.6%	99.7%
bvFTD	95%	88.9%	98.2%
EOAD	91.1%	87%	92.3%
LOAD	84.4%	78.1%	92.3%

The NR percentile results, including the cutoff, showed comparable results to other models in particular the NR index with predictive regions similar to the NR index (Model 4, Table 5).

**Table 5:** Model 4 NR percentile results

Classification method	% of original grouped cases correctly classified		% of cross-validated cases correctly classified
Discriminant analysis	89.1%		74.5%
**Automatic linear modeling predictor** **Region**			**Predictor importance**
Gray matter			0.23
White matter			0.10
Left frontal lobe			0.07
Right frontal lobe			0.06
Left lateral ventricle			0.06
Right ventral diencephalon			0.05
Left thalamus			0.05
Right lateral ventricle			0.05
Right pallidum			0.04
Left ventral diencephalon			0.04
**Diagnostic group**	**Sensitivity**	**Specificity**	**Area under the curve**
TBI	100%	100%	100%
bvFTD	100%	97.4%	99.7%
EOAD	93.3%	92.4%	97.2%
LOAD	93.8%	91.4%	96.8%
	**Sensitivity NR percentile cutoff ≤ 25th and ≥ 75th percentile for CSF Containing Structures**	**Specificity NR percentile cutoff ≤ 25th and ≥ 75th percentile for CSF Containing Structures**	**Area under the curve NR percentile cutoff ≤ 25th and ≥ 75th percentile for CSF Containing Structures**
TBI	97.5%	88.7%	97.4%
bvFTD	85%	82.9%	94.6%
EOAD	82.2%	83.6%	92.3%
LOAD	90.6%	85.7%	92.4%

The Z-score results in Model 5 are similar to that of the NR percentile and NR index (see Table 6).

**Table 6:** Model 5 Z-score Results

Classification method	% of original grouped cases correctly classified		% of cross-validated cases correctly classified
Discriminant analysis	91.2%		76.6%
**Automatic linear modeling predictor region**			**Predictor importance**
Gray matter			0.15
Left frontal lobe			0.07
Right frontal lobe			0.07
White matter			0.06
Whole brain matter (gray matter + white matter)			0.06
Right ventral diencephalon			0.05
Left thalamus			0.05
Right thalamus			0.04
Right lateral ventricle			0.04
Right pallidum			0.04
**Diagnostic group**	**Sensitivity**	**Specificity**	**Area under the curve**
TBI	100%	100%	100%
bvFTD	100%	97.4%	99.7%
EOAD	93.3%	92.4%	97.2%
LOAD	93.8%	91.4%	96.9%

## Discussion

This study investigated the diagnostic utility of MRI volumetry in differentiating between TBI, bvFTD, EOAD, and LOAD. Our findings demonstrate that volumetric measures, such as volume/mTIV, volume (ml), NR index, NR percentile, and Z-score, can effectively discriminate between these groups with high sensitivity, specificity, and accuracy. This work also highlights specific regional atrophy that helps to further delineate these disorders such as the parietal lobe in EOAD, the hippocampus in LOAD, the white matter in TBI, and the frontal lobes in bvFTD. These findings are important for clinicians seeing patients with neurocognitive disorders with differential considerations with similar symptoms wherein MRI is readily available thus highlighting the importance of quantitative software such as Neuroreader. This is especially important in clinical settings where fluid biomarkers and fluorodeoxyglucose positron emission tomography (FDG PET) are not readily available for patients.

Several discriminant models were developed to determine the performance of MR volumetry metrics in differentiating between the four groups. Model 1, which utilized volume/mTIV, yielded the highest cross-validated accuracy (80.3%), followed by Model 2 with volume (ml) (79.6%), Model 3 and 5 with NR index and Z-score (both 76.6%), and Models 4 with NR percentile (74.5%).

With respect to ROC analyses, the highest AUC was noted with TBI, followed by bvFTD, EOAD, and LOAD with results ranging from 96.8 to 100% for the NR index. Using a NR index of -2 or lower yielded comparable ranges of AUC results from 92.3% for LOAD to 99.7% for TBI. Thus, simple cutoffs can be applied for clinical use without loss of diagnostic performance.

The ROC curve sensitivity results of our study alone exceed those of visual scale, which range from between 80 and 85% for Alzheimer Disease [23–25]. With TBI, visual evaluations drop in effectiveness, seen only with 10% of CT scans and 30% of MRI scans [26–28]. These results are also improved upon by the ROC curve data for the TBI group from our study. For bvFTD, visual evaluations in one study had a sensitivity of 70% and specificity of 93% [29].

The results of our automatic linear modeling analyses revealed several key brain regions that contributed to the accurate classification of the four diagnostic groups. While some of the results are intuitive, such as the hippocampus being the lowest region in LOAD and one of the most diagnostically predictive, several results are of particular interest. For example, white matter atrophy in traumatic brain injury more so compared to the other diagnoses reflects the extent or previously reported abnormalities in both this diagnosis and tissue class [30]. Also, while both bvFTD and TBI had brain atrophy, there was a greater burden of frontal lobe volume loss in TBI reflected in our sample. However, caudate atrophy noted in our bvFTD sample is characteristic of the disorder [31] and can also related to motor system abnormalities in these patients [32]. EOAD showed a specific parietal lobe area of volume loss in keeping with our prior work [15]. Overall, our MRI volumetry results were strongest for traumatic brain injury.

Placing the results of our study in context with the diagnostic performance of existing FDA cleared clinical software programs, NeuroQuant, supplemented by NeuroGage that has additional asymmetry analyses, shows sensitivity of 100% and specificity of 95% for delineating TBI from non-TBI persons [33]. Another study in which NeuroQuant segmented hippocampus, lateral ventricles, and inferior lateral ventricles was applied to Dementia of Alzheimer Type demonstrated average AUC of 76%, sensitivity of 73% and specific of 71% [34]. Another FDA cleared program, AccuBrain, showed diagnostic utility in separating AD from FTD with AUC of 90%, sensitivity of 89% and specificity of 75% [35]. Another FDA cleared program, IcoMetrix, showed areas under the curve ranging from 90% for the hippocampus, 99% for the temporal lobes, and 89% for the lateral ventricles when separating AD persons from age matched controls, comparable to results from the research domain Freesurfer program [36]. However, these studies largely compared the neurocognitive conditions largely to normal controls and not to each other, a comparatively easier diagnostic question.

Additionally, the majority of FDA cleared programs lack diagnostic validation in dementia and non-dementia conditions. In prior work, of the 17 FDA cleared programs that existed at that time [11] only two of them – NeuroQuant and Neuroreader – had multiple publications with clinical validation in both dementia and non-dementia conditions. Several of the programs had no technical or clinical validation works published in peer-review. Thus, while our work advanced the field by testing FDA cleared software against harder diagnostic delineations, much additional work remains to apply this approach to similar such programs.

An additional strength of this study is the inclusion of well-characterized patient cohorts, who underwent extensive clinical evaluations and met the diagnostic criteria for TBI, bvFTD, EOAD, or LOAD. This may have contributed to the observed high sensitivity and specificity of the measures. However, we did not have biomarker confirmation of the AD and bvFTD diagnoses and thus do not have definitive confirmation of the neuropathological diagnoses. However, the clinical classification of AD shows high correlation with neuropathological results with a sensitivity of 98% and diagnostic accuracy of 88% though with a relatively lower specificity of 69% [37]. When comparing hippocampal and mesial temporal lobe volume loss to gold standard neuropathological diagnosis, MRI volumetry shows sensitivity ranging from 88 to 95% and specificity of 92–94% [38]. With FTD, clinical criteria for the diagnosis is 85% sensitive and 82% specific [39]. As TBI is purely a clinical diagnosis we are overall confident of our clinical diagnoses even with lack of biomarkers in several of the conditions we analyzed. However, as fluid biomarkers become of readily available for Alzheimer and related dementias future work should evaluate findings similar to ours in the context of biomarker confirmed diagnoses. While TBI severity was not available in this cohort our TBI participants with their underlying cognitive dysfunction are most likely to experience future dementia [40] and thus prone to develop similar atrophy patterns that we were able to delineate from the other clinical dementias in this study. However, as atrophy is more likely to be seen TBI with multiple traumatic events of increased time after injury, newer methods and sequences will be needed to improve diagnostic detection of TBI [41, 42]. Another weakness of the study was use of both 1.5 and 3.0T scanners as these field strengths were not the same in the study. A common reality of clinical practice is that identical field strengths may not always be available for these evaluations though every effort should be made to ensure so. However, in our study this weakness did not appear to reduce diagnostic performance for TBI in model 1 though it is possible it may have reduced or had no effect on diagnostic performance at all. This further suggested in prior work showing that tracking progression of AD atrophy on MR imaging is equivalent across 1.5T and 3.0T field strengths [43].

Our findings have several clinical implications. First, they support the use of MRI volumetry as an adjunct to clinical evaluation, biomarker testing, and neuropsychological testing in the diagnosis of TBI, bvFTD, EOAD, and LOAD. This may improve diagnostic accuracy and help guide appropriate treatment and management strategies for these patients and MRI remains a key part of standard dementia imaging evaluations [44, 45]. Second, the identification of key brain regions contributing to accurate group classification may inform future research on the underlying pathophysiology of these disorders and aid in the development of targeted interventions. Lastly, availability of MRI as a method for evaluating brain structure ensures that MRI volumetry will be quite impactful to patient care.

## Electronic supplementary material

Below is the link to the electronic supplementary material.

Supplementary Material 1

## Data Availability

No datasets were generated or analysed during the current study.
