# Supplementary material for: Diagnostic utility of brain MRI volumetry in comparing traumatic brain injury, Alzheimer disease and behavioral variant frontotemporal dementia
Source: BMC Neurol. 2024 Sep 11;24:337. doi: 10.1186/s12883-024-03844-4 (PMC11389120; doi:10.1186/s12883-024-03844-4)
Supplement: Supplementary file 1 — Supplementary Material 1 [file 12883_2024_3844_MOESM1_ESM.docx]

**Supplemental Table 1:** Brain Volume Differences Across Diagnostic Groups (TBI, bvFTD, EOAD, LOAD) compared to published Normative Values

| **Lowest Brain Volume or Largest CSF Containing Structure Across Diagnoses** | **ANOVA Results for Vol/mTIV (F-value, p-value)** |
| --- | --- |
| **TBI** | |
| White Matter | 5.8, .007 |
| Brainstem | 1234.6, < .001 |
| Left Cerebellum | 1471.1, < .001 |
| Right Cerebellum | 1452.3, < .001 |
| Left Frontal | 283.8, < .001 |
| Right Frontal | 284.9, < .001 |
| Right Parietal | 258.9, < .001 |
| **bvFTD** | |
| Gray Matter | 32.8, < .001 |
| CSF | 396.9, < .001 |
| Left Caudate | 1737.5, < .001 |
| Right Caudate | 684.2, < .001 |
| Left Putamen | 125.7, < .001 |
| Right Putamen | 7.2, < .001 |
| Left Thalamus | 834.6, < .001 |
| Right Thalamus | 3356.1, < .001 |
| Left Ventral Diencephalon | 3640.9, < .001 |
| Right Ventral Diencephalon | 3605.1, < .001 |
| Right Pallidum | 54.6, < .001 |
| **EOAD** | |
| Left Parietal Lobe | 9.4, < .001 |
| **LOAD** | |
| Whole Brain Matter | 11.6, < .001 |
| Left Hippocampus | 13.1, < .001 |
| Right Hippocampus | 7.3, < .001 |
| Left Temporal Lobe | 16.3, < .001 |
| Right Temporal Lobe | 6.6, < .001 |
| Left Amygdala | 13.5, < .001 |
| Right Amygdala | 5.5, < .001 |
| Left Lateral Ventricle | 52.3, < .001 |
| Right Lateral Ventricle | 56.2, < .001 |
| Right Occipital Lobe | 1503.5, < .001 |
| Left Pallidum | 57.2, < .001 |
